# Mexicanolide-Type Limonoids from the Roots of *Trichilia sinensis*

**DOI:** 10.3390/molecules21091152

**Published:** 2016-08-30

**Authors:** Shou-Bai Liu, Wen-Li Mei, Hui-Qin Chen, Zhi-Kai Guo, Hao-Fu Dai, Zhu-Nian Wang

**Affiliations:** 1 Tropical Crops Genetic Resources Institute, Chinese Academy of Tropical Agricultural Sciences, Danzhou 571737, China; zhiwu19831113@163.com; 2 Key Laboratory of Biology and Genetic Resources of Tropical Crops, Ministry of Agriculture, Institute of Tropical Bioscience and Biotechnology, Chinese Academy of Tropical Agricultural Sciences, Haikou 571101, China; meiwenli@itbb.org.cn (W.-L.M.); chenhuiqin@itbb.org.cn (H.-Q.C.); guozhikai@itbb.org.cn (Z.-K.G.)

**Keywords:** *Trichilia sinensis*, meliaceae, limonoids, mexicanolide-type, AChE inhibitory activity

## Abstract

Four new mexicanolide-type limonoids **1**–**4**, along with two known limonoids **5**–**6**, were isolated from the ethanolic extracts of roots of the Traditional Chinese Medicine *Trichilia sinensis*. Their structures were unambiguously determined by analysis of spectroscopic data, including 1D and 2D NMR as well as MS, and by comparison with literature data. In addition, the acetylcholinesterase (AChE) inhibitory activity of compounds **1**–**6** was evaluated by the Ellman method. All these compounds showed weak AChE inhibitory activity, with the inhibition percentages ranging from 18.5% to 27.8%.

## 1. Introduction

**Figure 1:**
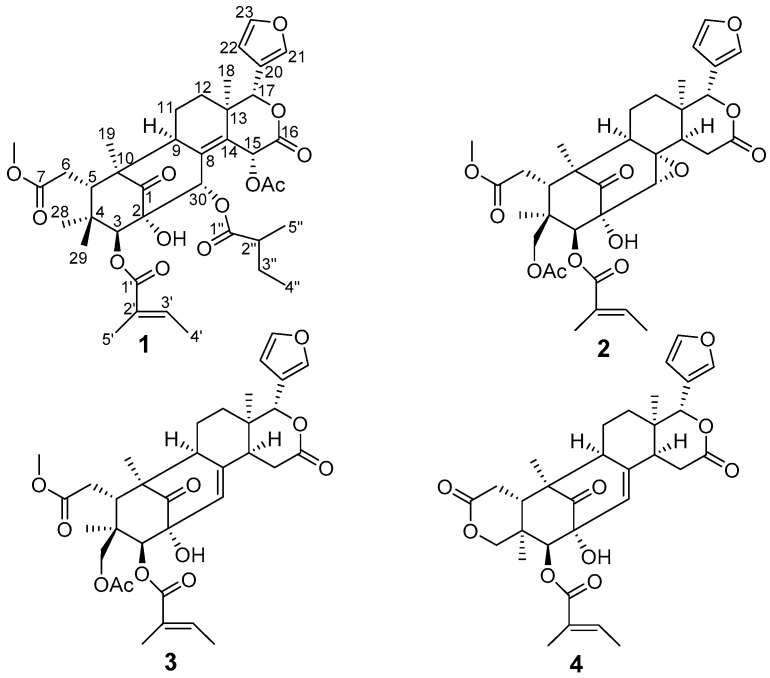
Structures of trichinenlides U–X (**1**–**4**).

Limonoids, as the major secondary metabolites of the Meliaceae family, are well-known for their abundance, structural diversity and a wide range of antifeedant, antimalarial, antimicrobial, cytotoxic, and growth-regulating activities [1,2]. The genus *Trichilia* of the Meliaceae consists of approximately 86 species, mainly distributed in the regions of tropical America, tropical Africa, India, Indochina, and the Malay Peninsula. Two species and one variant grow in China [3], and in folk medicine, *T. sinensis* Bentv. has traditional applications for the treatment of several diseases such as abdominal pain caused by *Ascaris lumbricoides*, chronic osteomyelitis, scabies, and eczema [4]. In Hainan Island, the roots and leaves of this plant are used by the local Li people to treat rheumatism and traumatic injury [5]. Previous phytochemical investigation on *T. sinensis* has led to the identification of a series of mexicanolide-type limonoids, some of which showed significant inhibition against lipopolysaccharide-induced nitric oxide production in RAW 264.7 macrophages [6]. During further investigation of limonoids from this plant, four new mexicanolide-type limonoids, named trichinenlides U–X (**1**–**4**, Figure 1) and two known ones **5**–**6**, were isolated from the EtOH extracts of the roots of *T. sinensis*. In this paper, the isolation and structure elucidation of the new compounds as well as their biological evaluation focused on acetylcholinesterase (AChE) inhibitory activity are reported.

## 2. Results and Discussion

Trichinenlide U (**1**), was obtained as a white amorphous powder, and a molecular formula of C_39_H_50_O_13_ was deduced from a HREIMS peak at *m/z* 726.3244 (calcd. 726.3251), indicating the presence of 15 degrees of unsaturation. The strong IR absorptions at 3443 and 1729 cm^−1^ implied the presence of hydroxyl and ester carbonyl groups, respectively. The ^1^H-NMR (Table 1) exhibited signals for nine methyl groups [δ_H_ 0.74 (3H, s), 0.78 (3H, s), 0.94 (3H, d, *J* = 7.5 Hz), 1.11 (3H, s), 1.17 (3H, d, *J* = 6.8 Hz), 1.28 (3H, s), 1.80 (3H, d, *J* = 6.8 Hz), 1.96 (3H, s) and 2.03 (3H, s)], one methoxy group [δ_H_ 3.74 (3H, s)], four oxymethine protons [δ_H_ 5.11 (1H, s), 5.68 (1H, s), 5.76 (1H, s) and 6.55 (1H, s)], as well as four olefinic protons [δ_H_ 6.52 (1H, s), 6.82 (1H, q, *J* = 6.8 Hz), 7.42 (1H, br s) and 7.65 (1H, s)]. The ^13^C-NMR displayed the corresponding carbons, in addition of four methylenes, four oxygenated methines and fourteen quarternary carbons (four olefinic, six carbonyls, and one oxygenated), as supported by DEPT and HSQC experiments. Further analysis of ^1^H- and ^13^C-NMR (Table 1 and Table 2), the structural framework of a mexicanolide-type limonoid was suggested for **1**, including the presence of a typical β-substituted furan ring (δ_H_ 6.52, 7.42, 7.65; δ_C_ 110.1, 120.8, 142.3, and 143.1), six membered δ-lactone (δ_H_ 5.68, 6.55; δ_C_ 39.3, 64.3, 80.4, 139.8 and 167.8), four tertiary methyls (δ_H_ 0.74, 0.78, 1.11 and 1.28; δ_C_ 17.3, 17.4, 19.5 and 23.7), one ketocarbonyl (δ_C_ 213.0), five ester carbonyls (δ_C_ 167.3, 167.8, 168.2, 174.2, and 174.3), and three additional ring derived from the hydrogen deficiency [7,8]. Extensive analysis of 2D NMR (^1^H-^1^H COSY and HMBC) spectra, a tigloyl moiety, an isobutyryl group, and one acetoxyl group were inferred.

**Figure 2:**
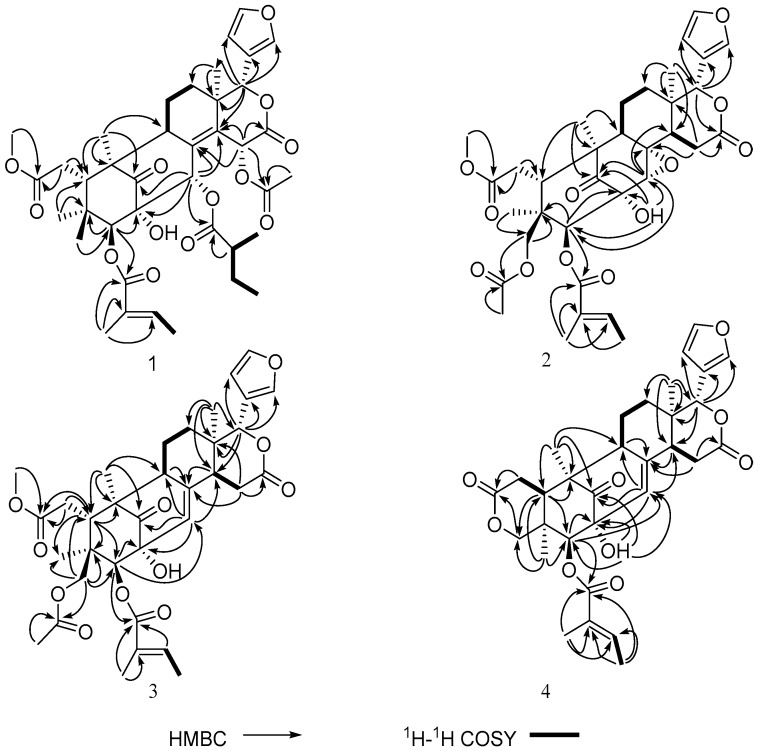
Selected ^1^H-^1^H COSY and HMBC correlations of **1**–**4**.

Comparison of the ^1^H- and ^13^C-NMR data (Table 1 and Table 2) of **1** showed it was structurally related to a known limonoid, trichinenlide L [6], the major difference between **1** and trichinenlide L being that the acetoxyl group at C-30 in trichinenlide L was replaced by an isobutyryl group in compound **1,** which was supported by the ^1^H-^1^H COSY correlation of H-2′′/H-5′′ and H-2′′/H-3′′/H-4′′, as well as the HMBC correlations of H-30/C-1′′, C-8, C-14, C-1, C-2 and H-2′′/C-1′′ (Figure 2).

HMBC correlations of H-3/C-1′, C-4, C-2, H_3_-29/C-3 and H-5′/C-1′, C-2′, C-3′ indicated that the tigloyl moiety was attached to C-3 (Figure 2). The position of acetoxyl moiety at C-15 was deduced by the HMBC correlations from H-15 to C-8, C-14, C-16 and the carbonyl of the acetyl group (Figure 2). Therefore, the planar structure of trichinenlide U (**1**) was elucidated as indicated.

**Figure 3:**
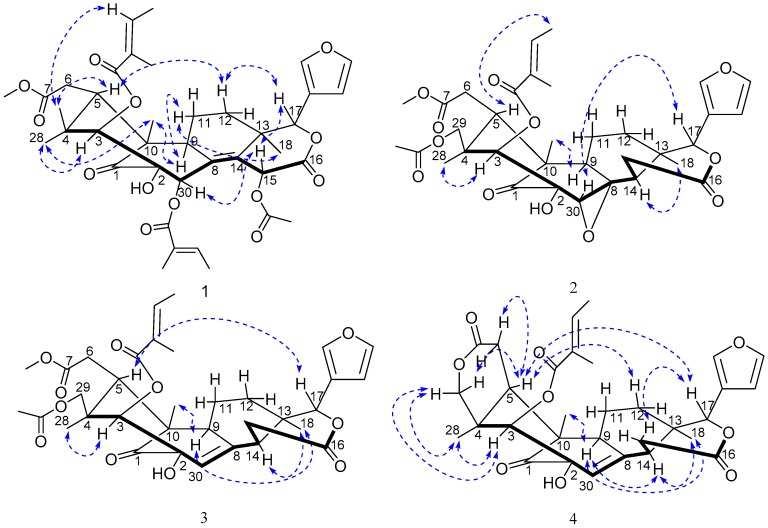
Key ROESY correlations of **1**–**4**.

The relative configuration of **1** was established by ROESY spectrum (Figure 3), in which the correlations of Me-29/H-5, Me-29/H-3ʹ, H-5/H-12β, H-12β/H-17, H-17/H-15, and H-15/H-30, indicated that these protons and the C-3 tigloyl group were arbitrarily assigned β-orientation. The ROESY cross-peaks of H-11α/Me-18, H-9/Me-19, Me-28/Me-19, Me-28/H-3, and H-9/H-11α revealed that all these protons were cofacial and located at α-face. Thus, **1** was identified as a new compound, and the trivial name of trichinenlide U was proposed.

Trichinenlide V (**2**), a white, amorphous powder, had the molecular formula C_34_H_42_O_12_, as established by a HRESIMS ion at *m*/*z* 665.2587 [M + Na]^+^ (calcd for C_34_H_42_O_12_Na, 665.2574), corresponding to 14 degrees of unsaturation. The IR absorptions indicated the presence of hydroxyl group (3431 cm^−1^) and carbonyl group (1735 cm^−1^). Detailed analysis of the ^1^H- and ^13^C-NMR spectra (Table 1 and Table 2) revealed the same mexicanolide-type limonoids skeleton with **1**, containing a furan ring [δ_H_ 6.43 (H, d, *J* = 1.0 Hz), 7.42 (H, t, *J* = 1.6 Hz), 7.49 (H, s); δ_C_ 110.3, 120.2, 141.1, 143.3], six member δ-lactone (δ_H_ 1.60, 2.78, 3.38, 5.17; δ_C_ 32.7, 36.4, 45.4, 78.8 and 171.3), C-7 carbomethoxy ester (δ_H_ 3.76; δ_C_ 52.8, 173.5), as well as the carbonyl group at C-1 (δ_C_ 212.5). By comparison, the structure of **2** were highly similar to that of heytrijunolide D [9]. The noticeable differences were the presence of a tigloyl unit at C-3 (δ_C_ 82.3) and an acetyl group at C-29 (δ_C_ 66.5) in **2**, which was confirmed by the cross-peaks in HMBC spectrum. The key HMBC correlations from H-3 to C-1′, C-4 and C-2 indicated that the tigloyl moiety was attached to C-3, while the correlations from H-29 to C-4 and the carbonyl of the acetyl group and from H_3_-28 to C-29 further confirmed the location of the acetyl moiety at C-29 (Figure 2). Thus, the planar structure of trichinenlide V (**2**) was elucidated as indicated.

The relative configuration of **2** was deduced from the analysis of its ROESY correlations. As shown in Figure 3, the observed correlations of Me-18/H-14, Me-19/H-9, Me-28/H-3, revealed that all these protons were cofacial and located at α-face. The ROESY cross-peaks of H-17/H-30, H-5/H-4' indicated that these protons and the C-3 tigloyl group were arbitrarily assigned β-orientation. Thus, **2** was identified as a new compound, and the trivial name of trichinenlide V was proposed.

Compound **3** was assigned a molecular formula of C_34_H_42_O_11_ by HRESIMS at *m*/*z* 649.2635 ([M + Na]^+^, calcd for C_34_H_42_O_11_Na, 649.2625). The ^1^H- and ^13^C-NMR spectra data (Table 1 and Table 2) revealed the characteristic C-NMR resonances of mexicanolide-type limonoids possessing a trisubstituted double bond, a tigloyloxy group, and one *O*-acetyl group, exhibited most of the structural features found in compound **2**, with the major difference being the presence of a trisubstituted double bond instead of a trisubstituted oxirane of compound **3**. The Δ^8,30^ double bond was confirmed by the HMBC correlations from H-30 to C-1, C-2, C-9 and C-14, from H-9 to C-8, from H-14 to C-8, as well as from H-15 to C-8 (Figure 2). The relative configuration of **3** was assigned to be the same as that of **2** based on their similar 1D NMR data and the ROESY correlations (Figure 3) (See the Supplementary Materials). Therefore, a trivial name of trichinenlide W was given to **3**.

Trichinenlide W (**4**) was obtained as a white, amorphous powder. The molecular formula C_31_H_36_O_9_ was established by HRESIMS *m*/*z* 575.2252 ([M + Na]^+^, calcd for C_31_H_36_O_9_Na, 575.2257), indicating that compound **4** had 14 degrees of unsaturation. The strong IR absorptions at 3447 and 1728 cm^−1^ showed the presence of hydroxyl and carbonyl groups, respectively. The ^1^H- and ^13^C-NMR spectra data (Table 1 and Table 2) indicated eight of the 14 degrees of unsaturation occupied by one carbonyl group, three ester functionalities, and four carbon-carbon double bonds, therefore, six rings were required in the structure. The 2D NMR analysis (^1^H-^1^H COSY, HSQC, HMBC) (Figure 2) suggested the presence of a ketone (δ_C_ 213.6), a tigloyloxy group, and a β-furanyl ring. The aforementioned structural characteristic suggested that **4** was a mexicanolide-type limonoid, and showed high similarity to those of godavarin A [10], with the major difference involving the additional 2-OH in **4**. The proton signal at δ_H_ 4.12 that did not show correlation with any carbon in the HSQC spectrum was assigned to hydroxyl group at C-2 (δ_C_ 77.1) by the HMBC correlations from 2-OH to C-1, C-2, C-3, and C-30 (Figure 2). The cyclization of 29-methyl and ester carbon (δ_C_ 169.8, C-7) via oxygen to form a δ-lactone ring was revealed by the key correlations of HMBC from two coupled and oxygenated protons [δ_H_ 4.24 (d, *J* = 11.8 Hz, H-29α), 3.92 (d, *J* = 11.8 Hz, H-29β)] to C-7 (Figure 2). The same relative configuration of **4** as the known godavarin A was determined by the ROESY correlations (Figure 3). The observed ROESY correlations of H-3/H_3_-29, H-3/H_3_-28α, H-9/H_3_-19, H-9/H-14, and H-14/H_3_-18 indicated that these protons were all α-orientation, whereas the correlations of H-5/H-6β, H-5/H-28β, H-5/H-11β, H-5/H-12β, H-5/H-17, and H-17/H-15β revealed their β-oriented. Based on the above results, the relative configuration of **4**, named trichinenlide W, was established as shown). Two known compounds, humilin B (**5**) [11] and trichinenlide S (**6**) [8] were identified by comparison of their spectroscopic data with the literature data.

The inhibitory activity against AChE of the six limonoids **1**–**6** was evaluated in vitro. All the limonoids **1**–**6** showed inhibitory activity against AChE with the inhibition percentage of 18.8%, 21.2%, 18.5%, 23.7%, 27.8%, and 20.8%, respectively, at the concentration of 50 mg/mL.

## 3. Experimental Section

### 3.1. General Procedures

The IR spectra were obtained on a Nicolet 380 FT-IR instrument from KBr pellets (Thermo, Pittsburgh, PA, USA). The UV spectra were measured on a Shimadzu UV-2550 spectrometer (Beckman, Brea, CA, USA). Optical rotation was recorded using a Rudolph Autopol III polarimeter (Rudolph Research Analytical, Hackettstown, NJ, USA). The NMR spectra were recorded on a Bruker AV-500 spectrometer (Bruker, Bremen, Germany), using TMS as an internal standard. The HR-EI-MS were recorded with a AutospecPremier (Waters, Milford, MA, USA). The HR-ESI-MS were measured with an Agilent G6230 TOF MS (Agilent Technologies, Palo Alto, CA, USA). Column chromatography was performed with silica gel (Marine Chemical Industry Factory, Qingdao, China), Sephadex LH-20 (Merck, Darmstadt, Germany) and RP-18 (Merck). TLC was performed with silica gel GF254 (Marine Chemical Industry Factory), and detected by spraying with 5% H_2_SO_4_–EtOH.

### 3.2. Plant Material

The roots of *T. sinensis* were collected in Wanning, Hainan Province, China, in November 2011. The plant was identified by Prof. Zhengfu Dai of Institute of Tropical Bioscience and Biotechnology, Chinese Academy of Tropical Agricultural Sciences. A voucher specimen (No. 20111120) was deposited at Institute of Tropical Bioscience and Biotechnology, Chinese Academy of Tropical Agricultural Sciences.

### 3.3. Extraction and Isolation

The air-dried and powdered roots of *T. sinensis* (13.2 kg) were extracted three times with 95% EtOH (60.0 L) at room temperature to afford a crude extract (450.0 g). The extract was then dissolved in water and partitioned with petroleum ether (PE) (3.0 L × 3), EtOAc (3.0 L × 3), *n*-BuOH (3.0 L × 3) to give three parts. The EtOAc portion (212.0 g) was subjected to silica gel column chromatography, eluted with PE–EtOAc (from 10:1 to 1:1) followed by CHCl_3_–MeOH (from 25:1 to 0:1), to yield ten major fractions (Fr.1–Fr.10). Fr.3 (35.0 g) was subjected to vacuum liquid chromatography on silica gel, eluted with a gradient of CHCl_3_–MeOH (from 1:0 to 20:1), to give four parts (Fr.3A-Fr.3D). Fr.3C (7.0 g) was applied to silica gel with CHCl_3_–EtOAc (20:1 to 1:1) as eluent, to give four fractions, Fr.3C1–Fr.3C4. Fr.3C1 (1.1 g) was separated on a column of Sephadex LH-20 eluting with CHCl_3_–MeOH (1:1) to obtain Fr.3C1C (320.0 mg), then **1** (9.6 mg) was yield by chromatography on a silica gel column, eluting with CHCl_3_–MeOH (100:1). By using the same purification procedures, Fr.3A (9.0 g) afforded **5** (15.0 mg). Fr.5 (9.0 g) was chromatographed on Sephadex LH-20 eluted with CHCl_3_–MeOH (1:1) to give ten fractions, Fr.5A–Fr.5J. Fr.5D (710.0 mg) was chromatographed on a silica gel column eluting with CHCl_3_-acetone (15:1 to 5:1) to obtain four fractions, Fr.5D1–Fr.5D4; then Fr.5D1 (81.0 mg) was separated on a silica gel column eluting with petroleum ether–ethyl acetate (5:2) to obtain **2** (15.3 mg). Fr.5D (470.0 mg) was subjected to a silica gel column (CHCl_3_–MeOH, 200:1 to 50:1) to obtain two subfractions, Fr.5D1–Fr.5D6, and then Fr.5D5 (30.3 mg) was subjected to a silica gel column (petroleum ether–ethyl acetate, 7:3) to yield **3** (8.0 mg). By using the same purification procedures, Fr.6 afforded **4** (10.0 mg), and Fr.4 afforded **6** (2.0 mg).

*Trichinenlide U* (**1**): white, amorphous powder; ${\left[\alpha \right]}_{D}^{26}$ = −32 (*c* 1.3, MeOH); UV (MeOH) λ_max_ (log ε) 240 (1.58) nm; IR (KBr) ν_max_ 3444, 2925, 1729, 1635, 1469, 1261 cm^−1^; ^1^H- and ^13^C-NMR data: Table 1 and Table 2; HREIMS *m/z* 726.3244 (calcd for C_39_H_50_O_13_, 726.3251).

*Trichinenlide V* (**2**): white, amorphous powder; ${\left[\alpha \right]}_{D}^{26}$ = −98 (*c* 0.6, MeOH); UV (MeOH) λ_max_ (log ε) 231 (3.44) nm; IR (KBr) ν_max_ 3431, 2924, 1735, 1633, 1383, 1030 cm^−1^; ^1^H- and ^13^C-NMR data: Table 1 and Table 2; HRESIMS *m/z* 665.2587 (calcd for C_34_H_42_O_12_Na, 665.2574).

*Trichinenlide W* (**3**): white, amorphous powder; ${\left[\alpha \right]}_{D}^{26}$ = −236 (*c* 1.1, MeOH); UV (MeOH) λ_max_ (log ε) 229 (3.82) nm; IR (KBr) ν_max_ 3445, 2925, 1729, 1643, 1384, 1234, 1070 cm^−1^; ^1^H- and ^13^C-NMR data: Table 1 and Table 2; HRESIMS *m/z* 649.2635 (calcd for C_34_H_42_O_11_Na, 649.2625).

*Trichinenlide X* (**4**): white, amorphous powder; ${\left[\alpha \right]}_{D}^{26}$ = +151 (*c* 1.2, MeOH); UV (MeOH) λ_max_ (log ε) 215 (3.86) nm; IR (KBr) ν_max_ 3447, 2924, 1728, 1644, 1251, 1047 cm^−1^; ^1^H- and ^13^C-NMR data: Table 1 and Table 2; HRESIMS *m/z* 575.2252 (calcd for C_31_H_36_O_9_Na, 575.2257).

### 3.4. Bioassay of AChE Inhibitory Activity

Acetylcholinesterase inhibitory activity was assayed by the spectrophotometric method developed by Ellman with slightly modification. *S*-Acetylthiocholine iodide, 5,5′-dithio-bis-(2-nitrobenzoic) acid (DTNB), Ellman’s reagent and AChE were purchased from Sigma Chemical company (St. Louis, MO, USA). The specific experimental procedures were the same as those described previously [12].

## 4. Conclusions

The compounds **1**–**6** were characterized as trichinenlide U (**1**), trichinenlide V (**2**), trichinenlide W (**3**), trichinenlide X (**4**), humilin B (**5**) and trichinenlide S (**6**), respectively. To the best of our knowledge, so far, among these mexicanolide-type limonoids, the C-29 acetylation in compounds **2** and **3** is being reported for the first time. Compounds **1**–**4** were new limonoids, while **5** was isolated for the first time from the plant *T. sinensis*. All of the compounds showed weak inhibition against AChE.

## Figures and Tables

**Table 1:** ^1^H-NMR (500 MHz) data of compounds **1**–**4** (CDCl_3_, δ_H_ in ppm, *J* in Hz).

Proton	1	2	3	4
3	5.11 (s)	5.23 (s)	5.03 (s)	5.11 (s)
5	3.38 (dd, 10.0, 2.3)	3.38 (d, 1.4, 9.3)	3.51 (d, 10.1)	3.08 (dd, 10.9, 8.0)
6a	2.31 (m)	2.43 (m)	2.51 (dd,17.3, 10.3)	2.63 (m)
6b	2.42 (m)	2.43 (m)	2.40 (d, 17.3)	2.61 (m)
9	2.41 (m)	1.94 (m)	2.24 (m)	2.32 (m)
11α	1.77 (m)	1.80 (m)	1.66 (dd, 4.0, 13.2)	1.72 (m)
11β	1.90 (m)	1.97 (m)	2.02 (m)	1.79 (m)
12α	1.14 (m)	1.24 (m)	1.41 (m)	1.50 (m)
12β	1.85 (m)	1.94 (m)	1.61 (m)	1.69 (m)
14		1.60 (dd, 13.4, 5.1)	2.22 (m)	2.27 (m)
15α		3.38 (m)	2.84 (dd, 18.7, 6.1)	2.85 (m)
15β	6.55 (s)	2.78 (dd, 16.2, 5.1)	2.77 (d, 18.7)	2.85 (m)
17	5.68 (s)	5.17 (s)	5.56 (s)	5.37 (s)
18	1.11 (s)	0.99 (s)	1.09 (s)	1.02 (s)
19	1.28 (s)	1.20 (s)	1.27 (s)	1.24 (s)
21	7.65 (s)	7.49 (s)	7.81 (s)	7.46 (br s)
22	6.52 (s)	6.43 (d, 1.0)	6.46 (s)	6.36 (br s)
23	7.42 (br s)	7.42 (t, 1.6)	7.43 (s)	7.45 (br s)
28	0.74 (s)	0.86 (s)	0.93 (s)	1.03 (3H, s)
29a	0.78 (s)	3.87 (d, 10.8)	3.95 (d, 10.6)	3.92 (d, 11.8)
29b		3.73 (d, 10.8)	3.73 (d, 10.6)	4.24 (d, 11.8)
30	5.76 (s)	3.42 (s)	5.31 (s)	5.40 (s)
MeO-7	3.74 (s)	3.76 (s)	3.77 (s)	
3′	6.82 (q, 6.8)	7.00 (dq, 7.0, 1.3)	6.94 (q, 7.0)	6.86 (q, 6.9)
4′	1.80 (d, 6.8)	1.89 (d, 7.0)	1.72 (d, 7.0)	1.77 (d, 6.9)
5′	2.03 (s)	1.93 (s)	1.82 (s)	1.84 (s)
2′′	2.18 (m)			
3′′	1.77 (m)			
	1.49 (m)			
4′′	0.94 (t, 7.5)			
5′′	1.17 (d, 6.8)			
OAc-15	1.96 (s)			
OAc-29		1.93 (s)	1.95 (s)	
OH-2		4.06 (s)	4.17 (s)	4.12 (s)

**Table 2:** ^13^C-NMR (125 MHz) data of compounds **1**–**4** (CDCl_3_, δ_C_ in ppm).

Carbon	1	2	3	4
1	213.0	212.5	214.2	213.6
2	79.3	78.4	77.3	77.1
3	85.8	82.3	82.0	82.9
4	39.9	42.9	42.1	37.1
5	41.4	40.3	39.6	38.7
6	33.2	33.6	32.4	31.5
7	174.3	173.5	173.4	169.8
8	133.0	63.1	137.4	137.4
9	47.9	55.3	56.8	56.4
10	52.0	48.9	49.2	48.5
11	18.6	19.5	20.6	21.5
12	28.8	33.4	34.4	34.1
13	39.3	36.4	37.0	36.9
14	139.8	45.4	45.1	44.9
15	64.3	32.7	29.7	29.6
16	167.8	171.3	168.6	168.3
17	80.4	78.8	76.5	76.9
18	17.4	26.0	21.7	21.5
19	17.3	16.6	16.1	15.9
20	120.8	120.2	120.6	120.8
21	142.3.	141.1	142.1	141.2
22	110.1	110.3	109.8	109.4
23	143.1	143.3	143.3	143.6
28	23.7	14.9	14.9	16.4
29	19.5	66.5	66.7	73.7
30	74.2	67.4	129.0	128.5
MeO-7	52.5	52.8	52.7	
1′	167.3	166.3	167.0	166.9
2′	131.0	127.6	127.3	127.1
3′	137.4	140.4	140.5	140.5
4′	14.6	12.6	14.8	14.9
5′	12.9	15.8	11.6	12.2
1′′	174.2			
2′′	40.5			
3′′	26.6			
4′′	11.5			
5′′	14.5			
OAc-15	168.2			
	21.0			
OAc-29		170.5	170.6	
		20.6	20.7	

## Supplementary Materials

Supplementary materials can be accessed at: http://www.mdpi.com/1420-3049/21/9/1152/s1. The original spectra of NMR and positive-mode HRESIMS or HREIMS data for the new compounds (**1**–**4**) are available as Supplementary Materials.
