# Supplementary material for: Mexicanolide-Type Limonoids from the Roots of Trichilia sinensis
Source: Molecules. 2016 Aug 30;21(9):1152. doi: 10.3390/molecules21091152 (PMC6273291; doi:10.3390/molecules21091152)
Supplement: Supplementary file 1 [file molecules-21-01152-s001.pdf]

Supplementary Materials: Mexicanolide-Type Limonoids from the Roots of *Trichilia sinensis*

Shou-Bai Liu, Wen-Li Mei, Hui-Qin Chen, Zhi-Kai Guo, Hao-Fu Dai and Zhu-Nian Wang

Contents

Figure S1. ^{1}H-NMR (500 MHz, $CDCl_3$) spectrum of compound **1**
Figure S2. ^{13}C-NMR (125 MHz, $CDCl_3$) spectrum of compound **1**
Figure S3. HSQC spectrum of compound **1** in $CDCl_3$
Figure S4. ^{1}H-^{1}H COSY spectrum of compound **1** in $CDCl_3$
Figure S5. HMBC spectrum of compound **1** in $CDCl_3$
Figure S6. ROESY spectrum of compound **1** in $CDCl_3$
Figure S7. HREI-MS spectrum of compound **1**
Figure S8. ^{1}H-NMR (500 MHz, $CDCl_3$) spectrum of compound **2**
Figure S9. ^{13}C-NMR (125 MHz, $CDCl_3$) spectrum of compound **2**
Figure S10. HSQC spectrum of compound **2** in $CDCl_3$
Figure S11. ^{1}H-^{1}H COSY spectrum of compound **2** in $CDCl_3$
Figure S12. HMBC spectrum of compound **2** in $CDCl_3$
Figure S13. ROESY spectrum of compound **2** in $CDCl_3$
Figure S14. HRESI-MS spectrum of compound **2**
Figure S15. ^{1}H-NMR (500 MHz, $CDCl_3$) spectrum of compound **3**
Figure S16. ^{13}C-NMR (125 MHz, $CDCl_3$) spectrum of compound **3**
Figure S17. HSQC spectrum of compound **3** in $CDCl_3$
Figure S18. ^{1}H-^{1}H COSY spectrum of compound **3** in $CDCl_3$
Figure S19. HMBC spectrum of compound **3** in $CDCl_3$
Figure S20. ROESY spectrum of compound **3** in $CDCl_3$
Figure S21. HRESI-MS spectrum of compound **3**
Figure S22. ^{1}H-NMR (500 MHz, $CDCl_3$) spectrum of compound **4**
Figure S23. ^{13}C-NMR (125 MHz, $CDCl_3$) spectrum of compound **4**
Figure S24. HSQC spectrum of compound **4** in $CDCl_3$
Figure S25. ^{1}H-^{1}H COSY spectrum of compound **4** in $CDCl_3$
Figure S26. HMBC spectrum of compound **4** in $CDCl_3$
Figure S27. ROESY spectrum of compound **4** in $CDCl_3$
Figure S28. HRESI-MS spectrum of compound **4**

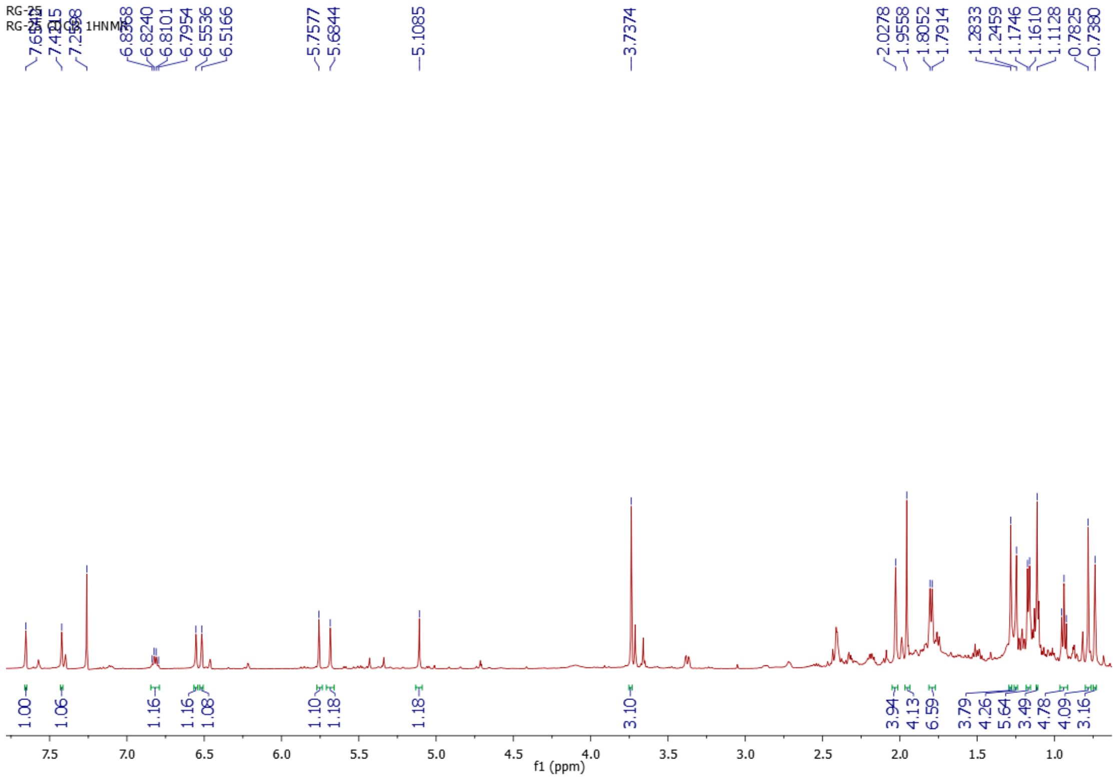

Figure S1. ^{1}H-NMR (500 MHz, $CDCl_3$) spectrum of compound **1**.

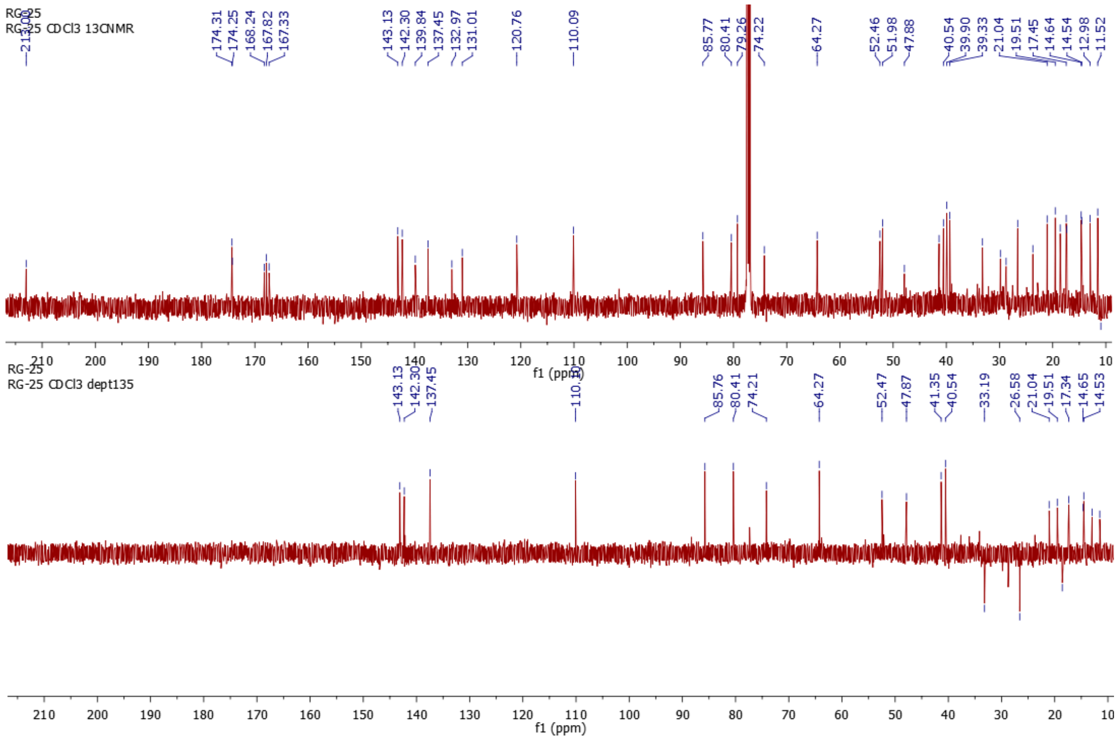

Figure S2. ^{13}C-NMR (125 MHz, $CDCl_3$) spectrum of compound **1**.

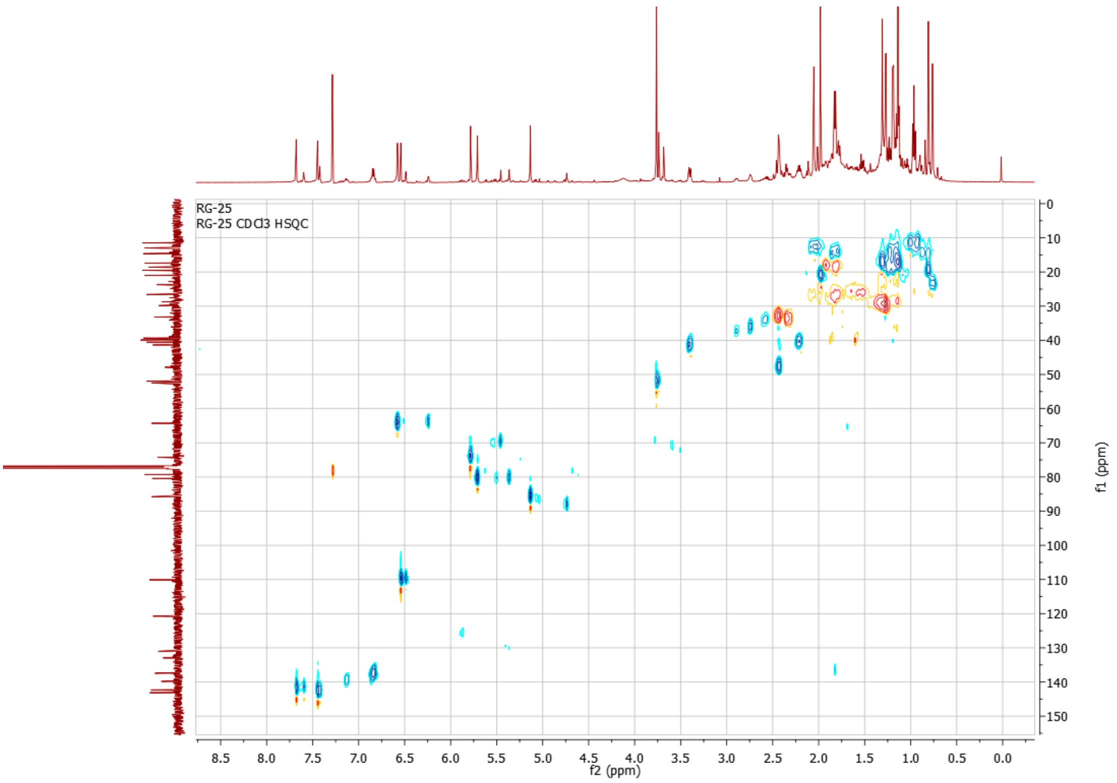

Figure S3. HSQC spectrum of compound **1** in $CDCl_3$.

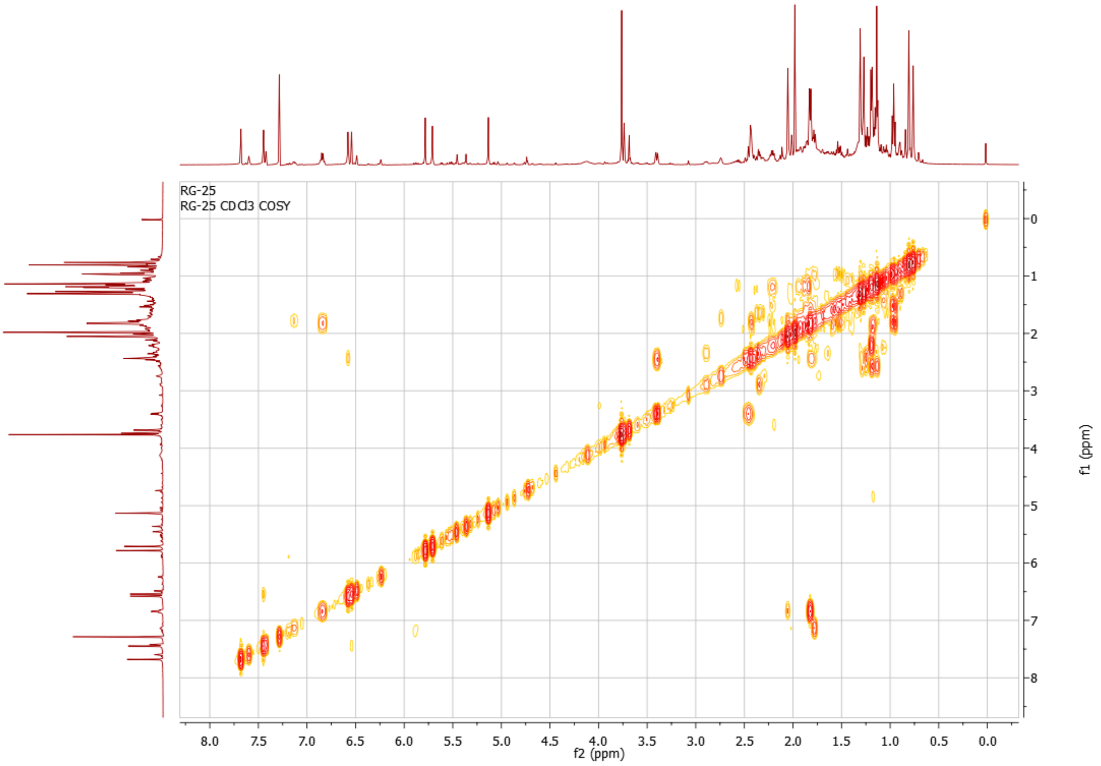

Figure S4. ^1H-^1H COSY spectrum of compound **1** in $CDCl_3$.

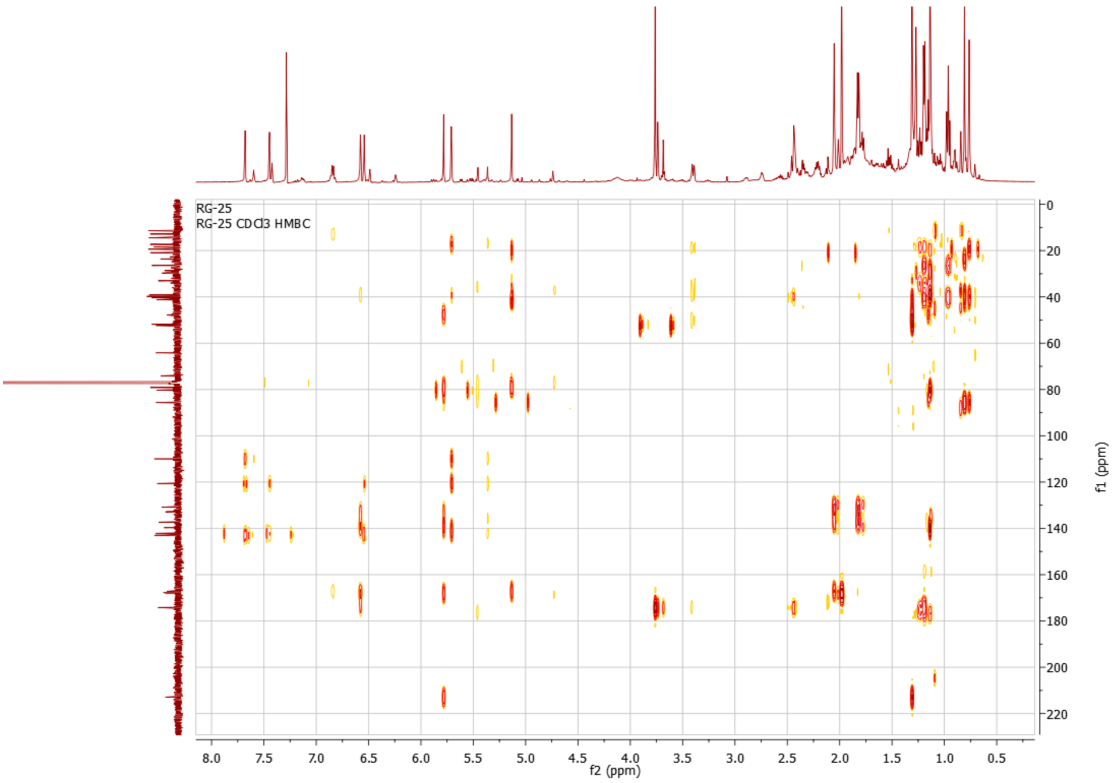

Figure S5. HMBC spectrum of compound **1** in $CDCl_3$.

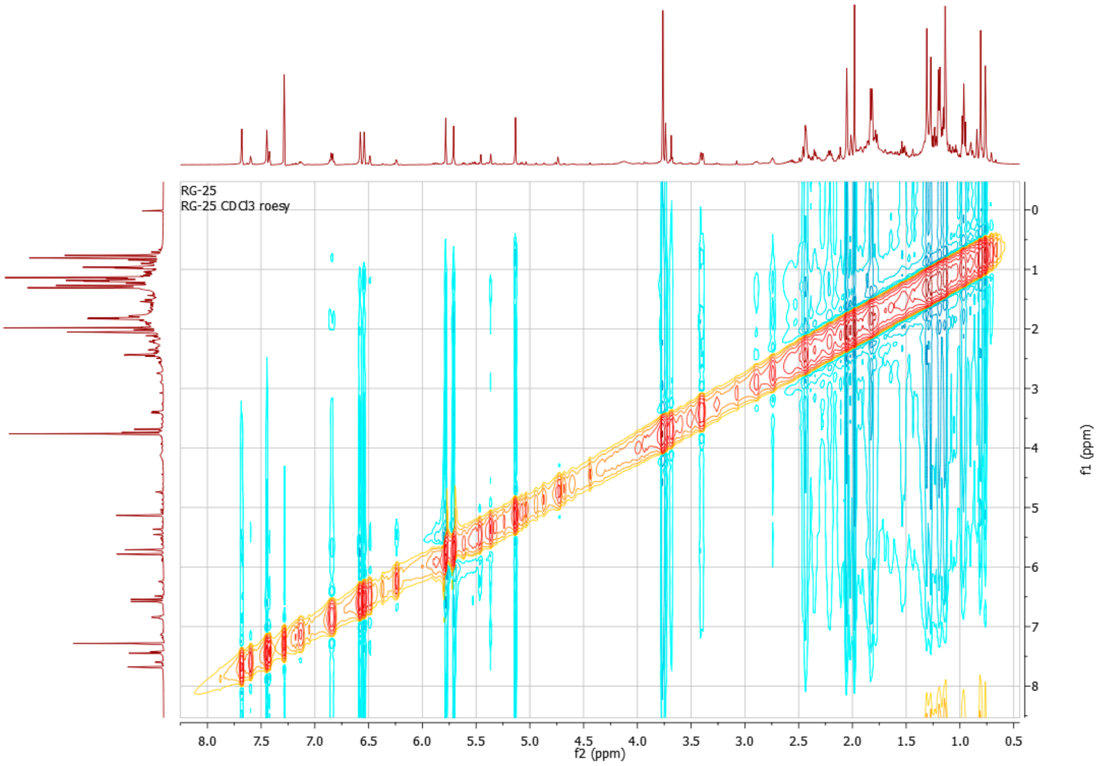

Figure S6. ROESY spectrum of compound **1** in $CDCl_3$.

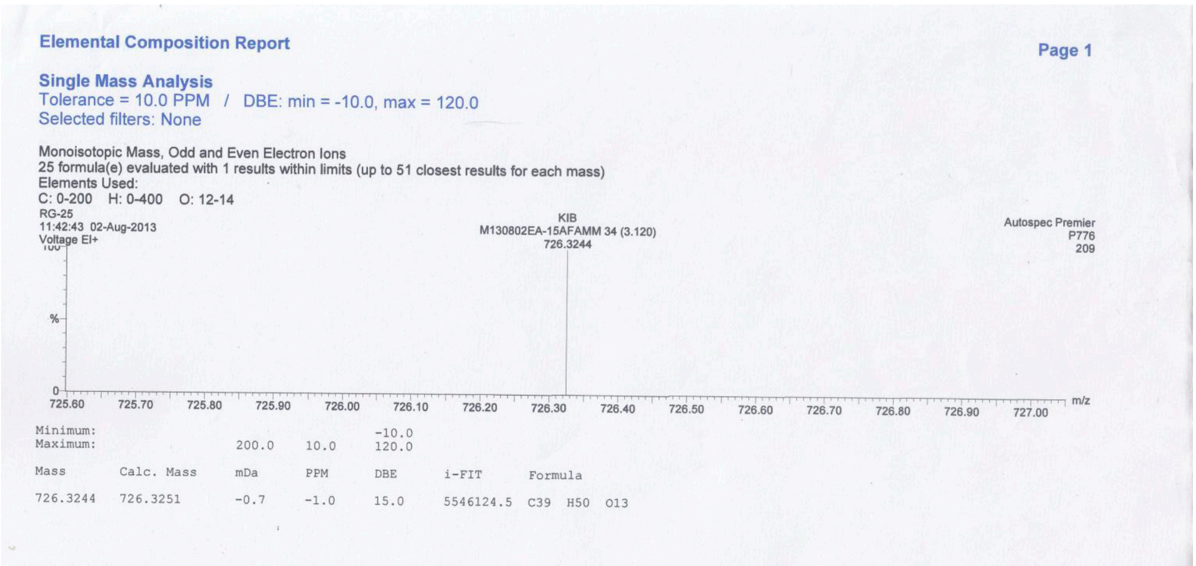

Figure S7. HREI-MS spectrum of compound **1**.

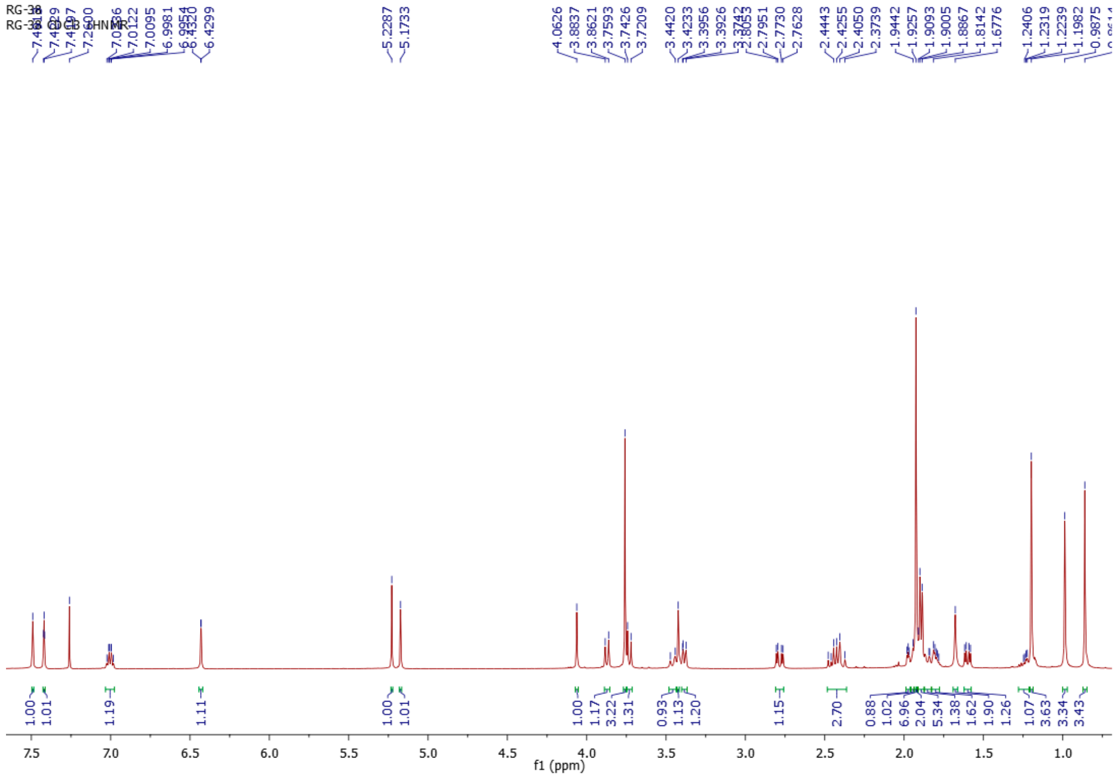

Figure S8. ^{1}H-NMR (500 MHz, $CDCl_3$) spectrum of compound **2**.

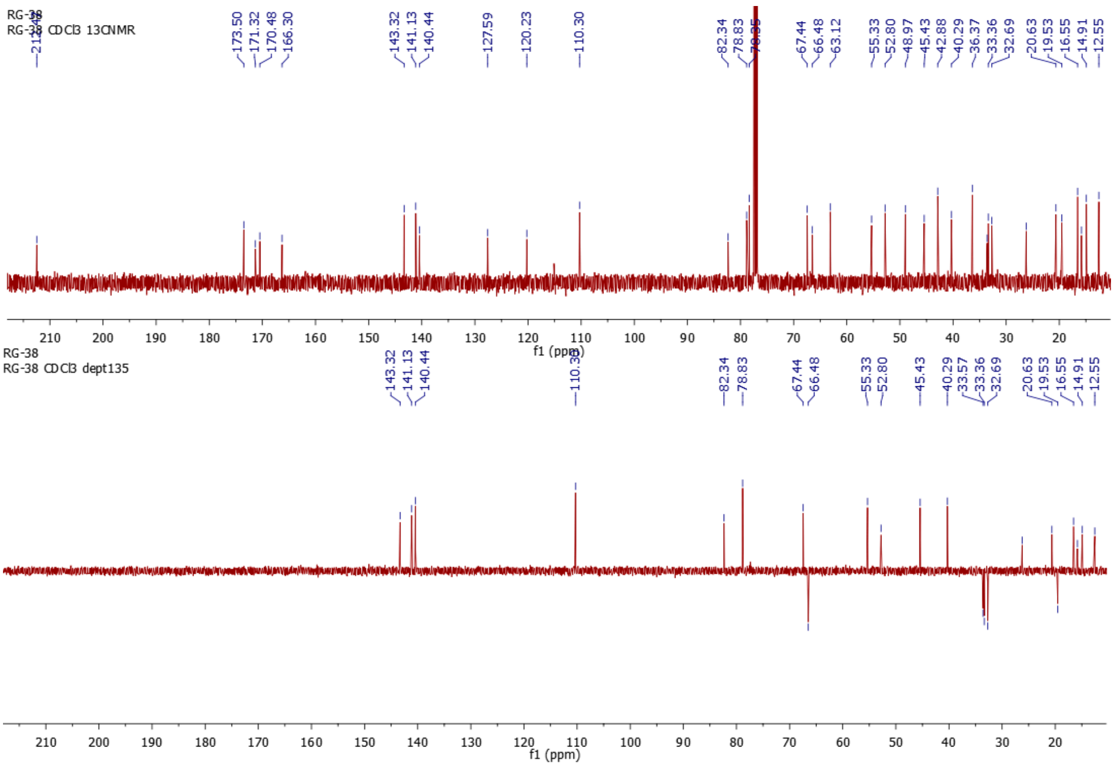

Figure S9. ^{13}C-NMR (125 MHz, $CDCl_3$) spectrum of compound **2**.

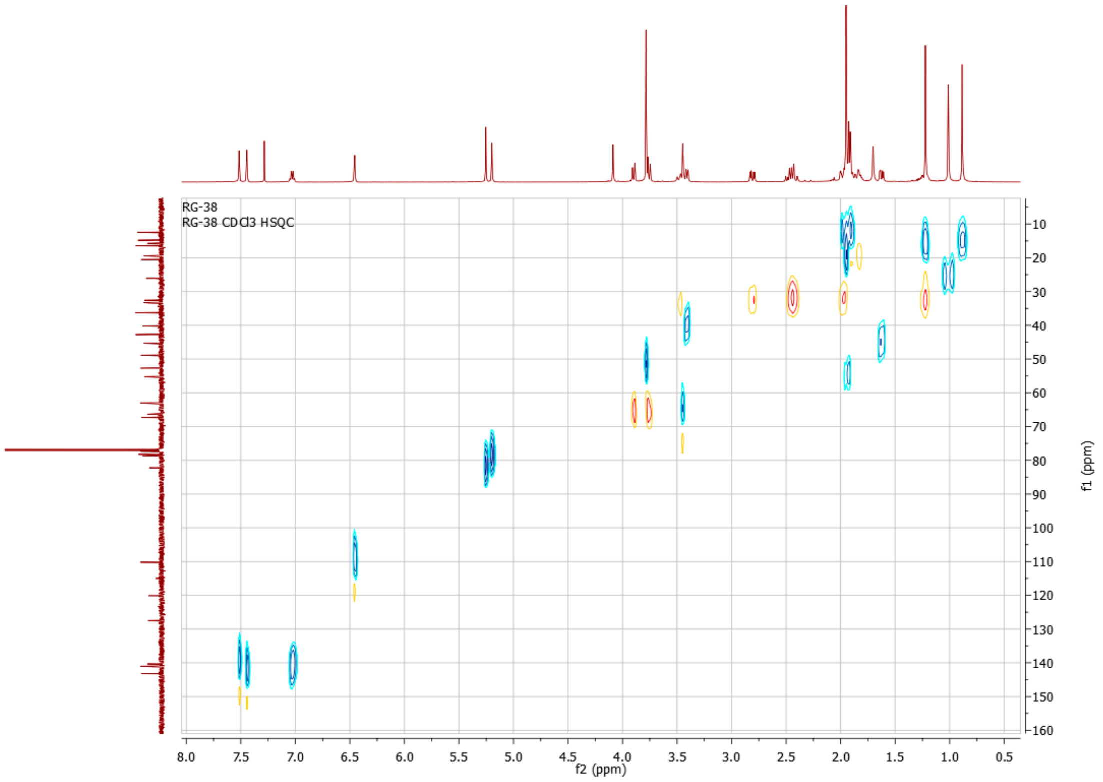

Figure S10. HSQC spectrum of compound **2** in $CDCl_3$.

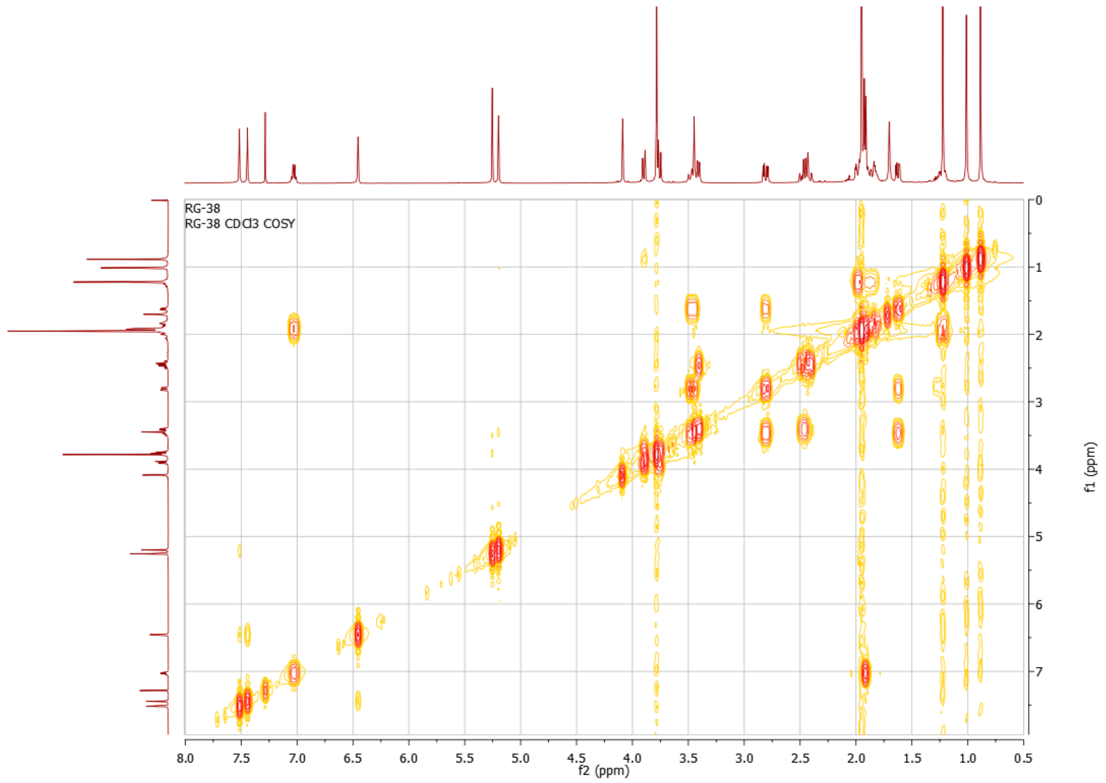

Figure S11. ¹H-¹H COSY spectrum of compound **2** in $CDCl_3$.

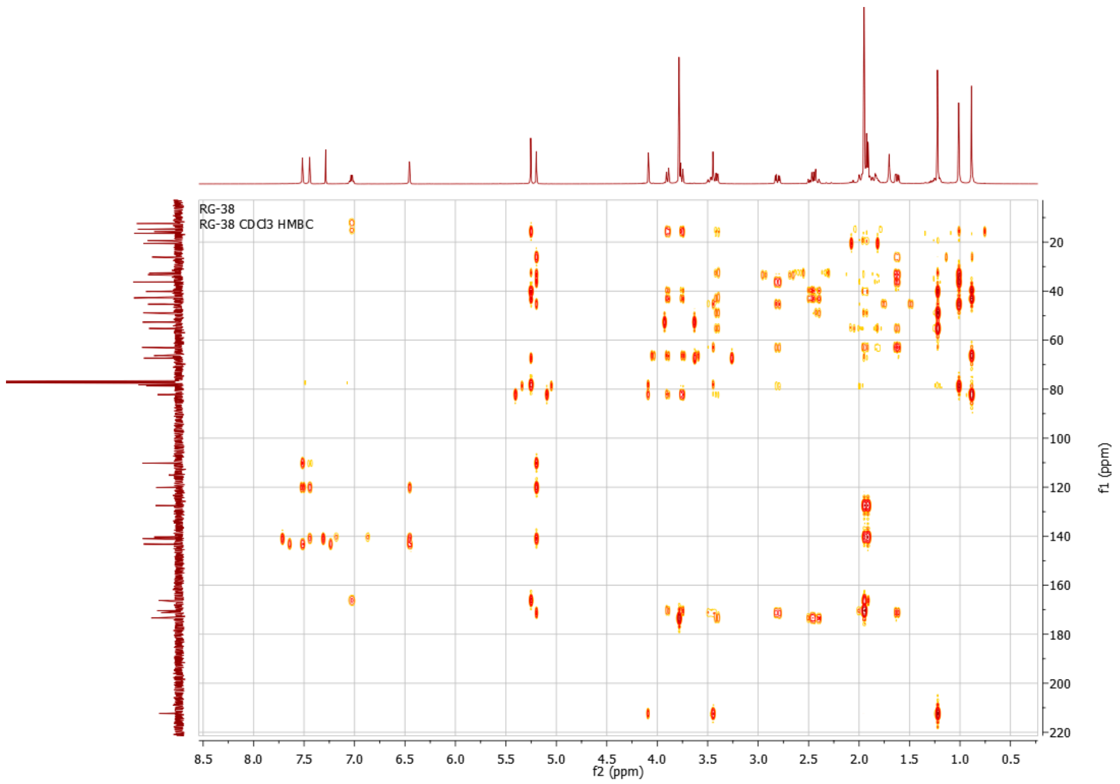

Figure S12. HMBC spectrum of compound **2** in $CDCl_3$.

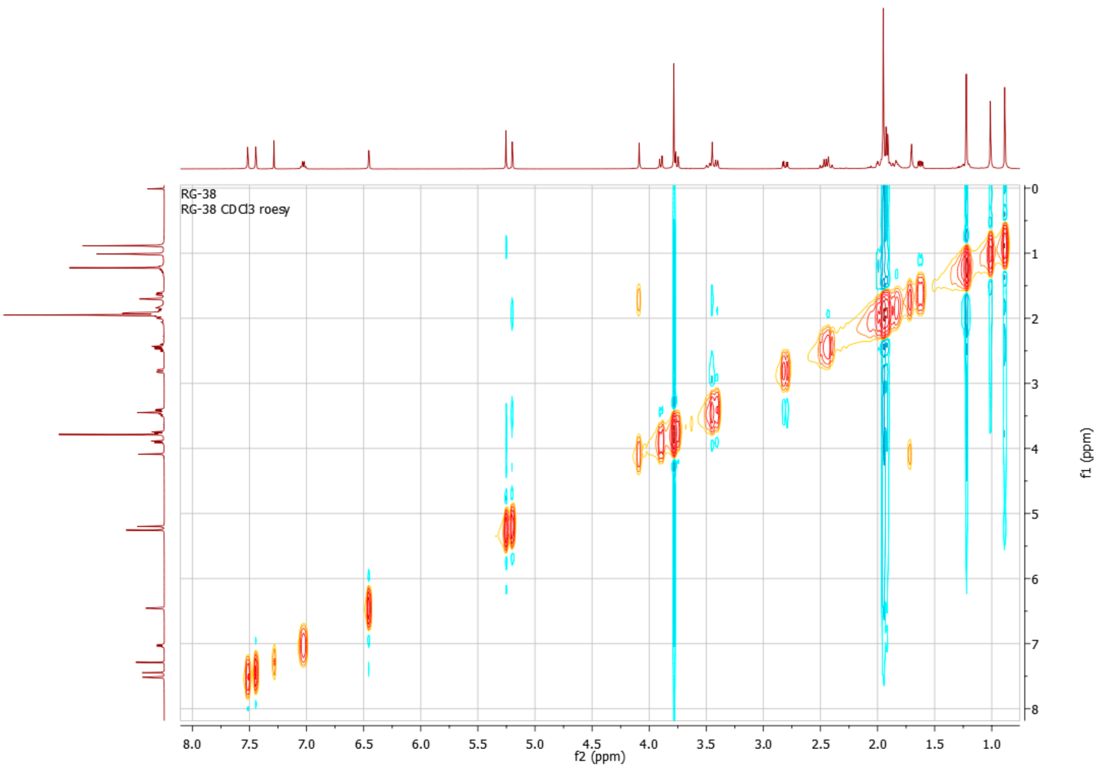

Figure S13. ROESY spectrum of compound **2** in $CDCl_3$.

Qualitative Analysis Report

**Data Filename**	140402ESIA5.d	**Sample Name**	RG-38
**Sample Type**	Sample	**Position**	
**Instrument Name**	Agilent G6230 TOF MS	**User Name**	KIB
**Acq Method**	ESI.m	**Acquired Time**	4/2/2014 11:36:35 AM
**IRM Calibration Status**	Success	**DA Method**	ESIN.m
**Comment**			

Sample Group **Info.**
Acquisition SW 6200 series TOF/6500 series
Version Q-TOF B.05.01 (B5125.1)

User Spectra

Fragmentor Voltage	Collision Energy	Ionization Mode
200	0	ESI

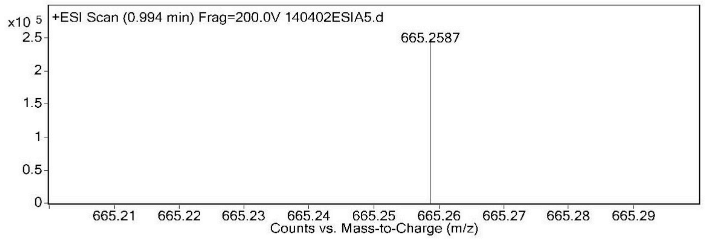

Peak List

m/z	z	Abund
1307.5271	1	360673.25

Formula Calculator Element Limits

Element	Min	Max
C	0	200
H	0	400
O	10	14
Na	1	1

Formula Calculator Results

Formula	CalculatedMass	Mz	Diff.(mDa)	Diff. (ppm)	DBE
C34 H42 Na O12	665.2574	665.2587	-1.3	2.0	13.5

--- End Of Report ---

Figure S14. HRESI-MS spectrum of compound **2**.

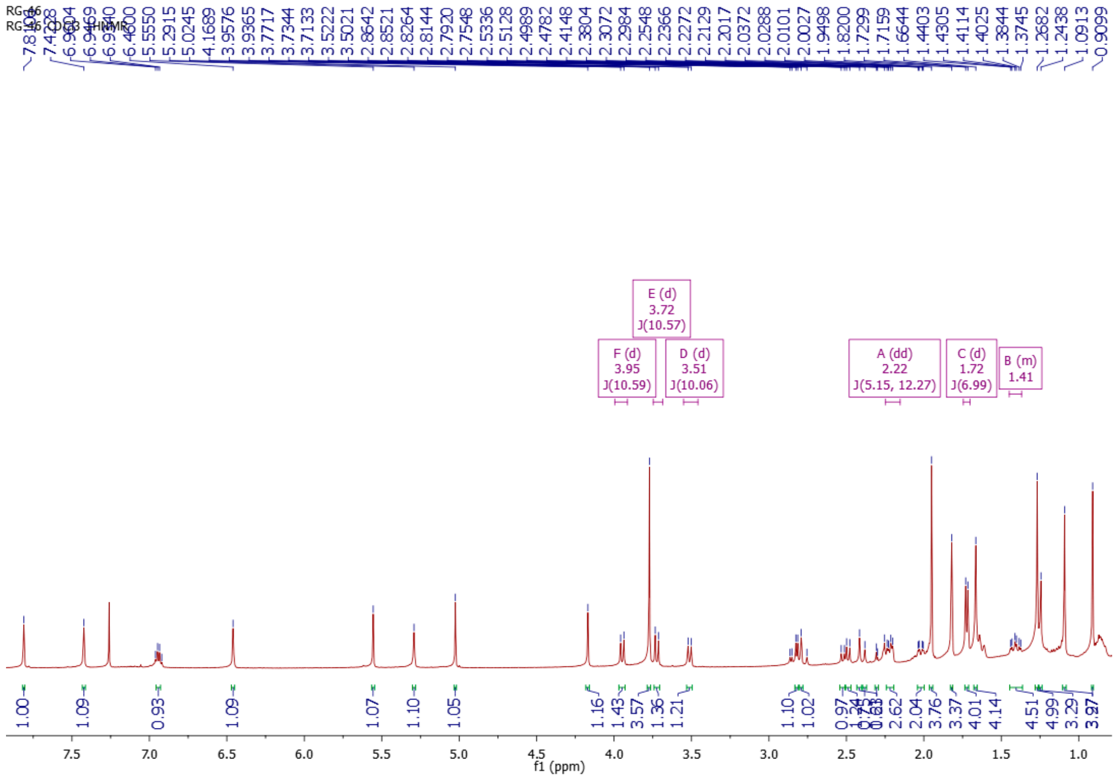

Figure S15. ^{1}H-NMR (500 MHz, $CDCl_3$) spectrum of compound **3**.

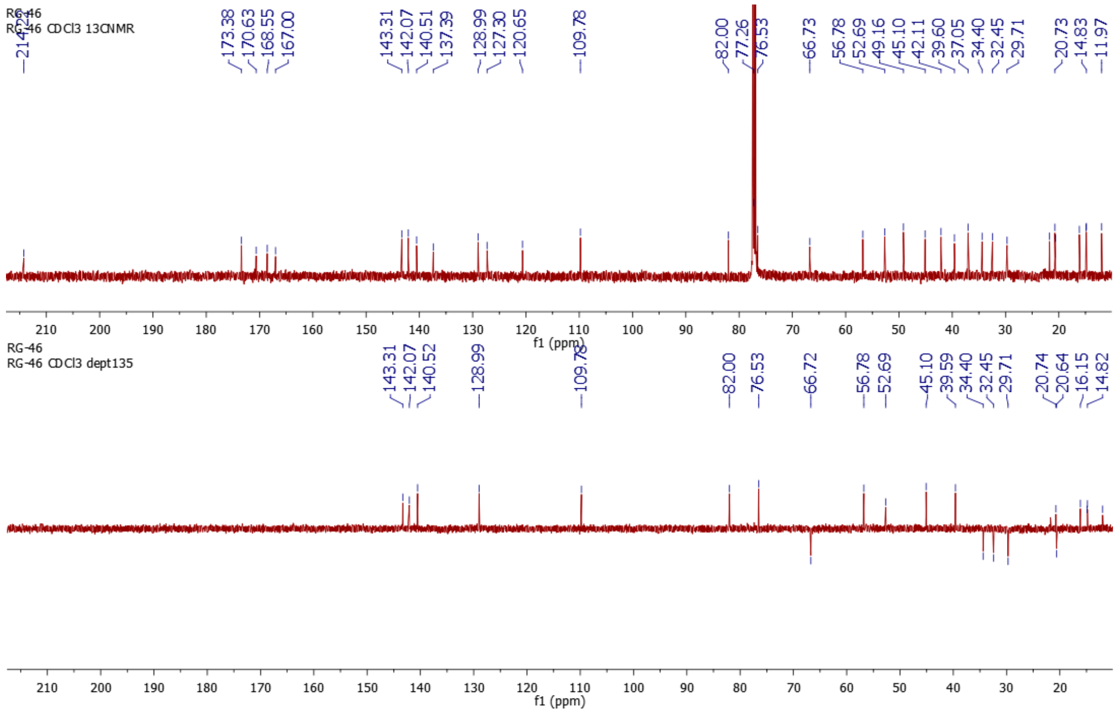

Figure S16. ^{13}C-NMR (125 MHz, $CDCl_3$) spectrum of compound **3**.

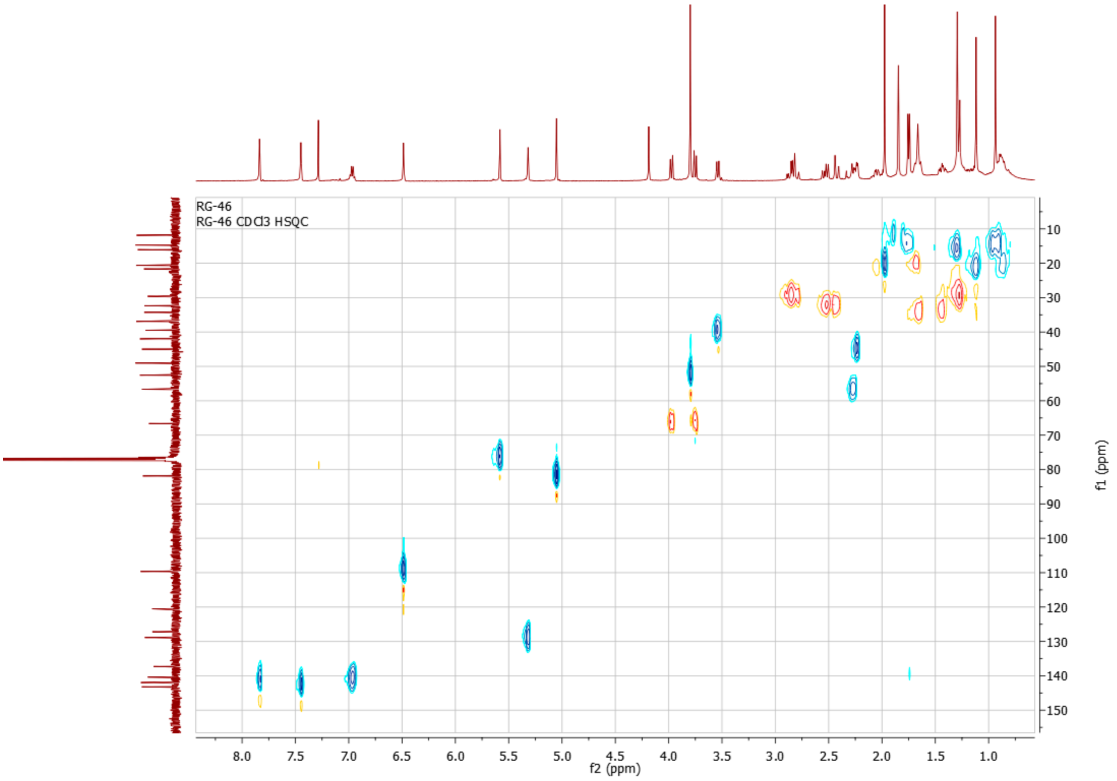

Figure S17. HSQC spectrum of compound **3** in $CDCl_3$.

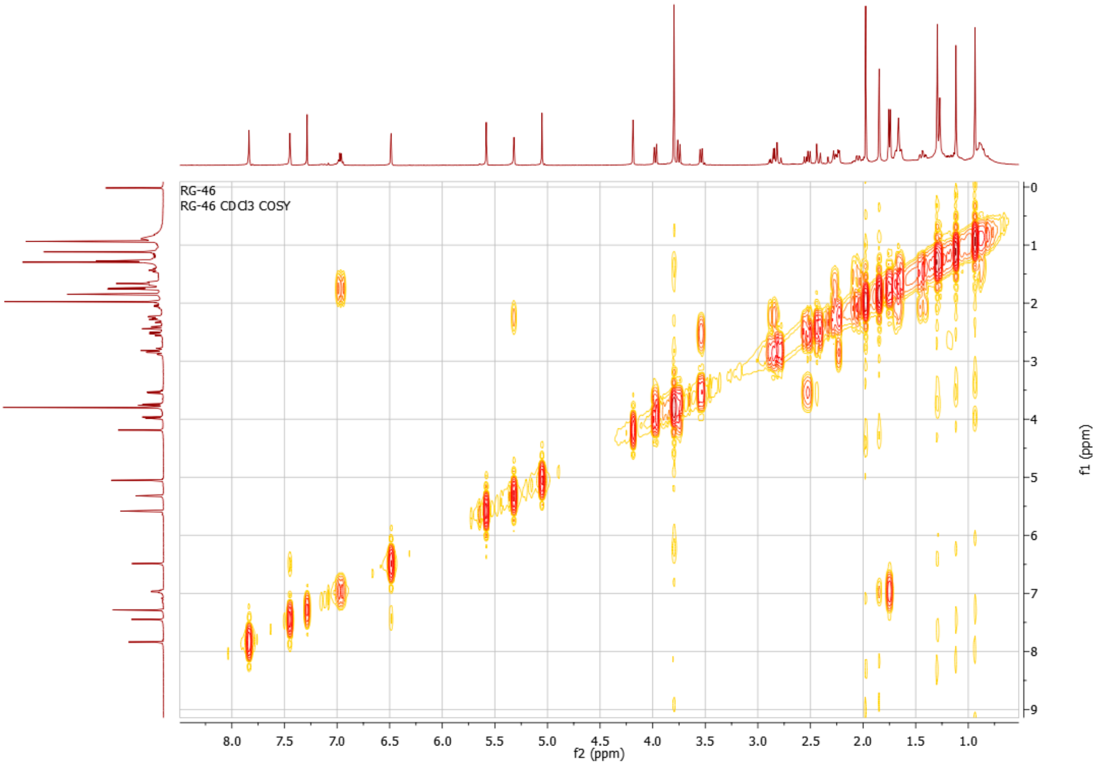

Figure S18. ^1H-^1H COSY spectrum of compound **3** in $CDCl_3$.

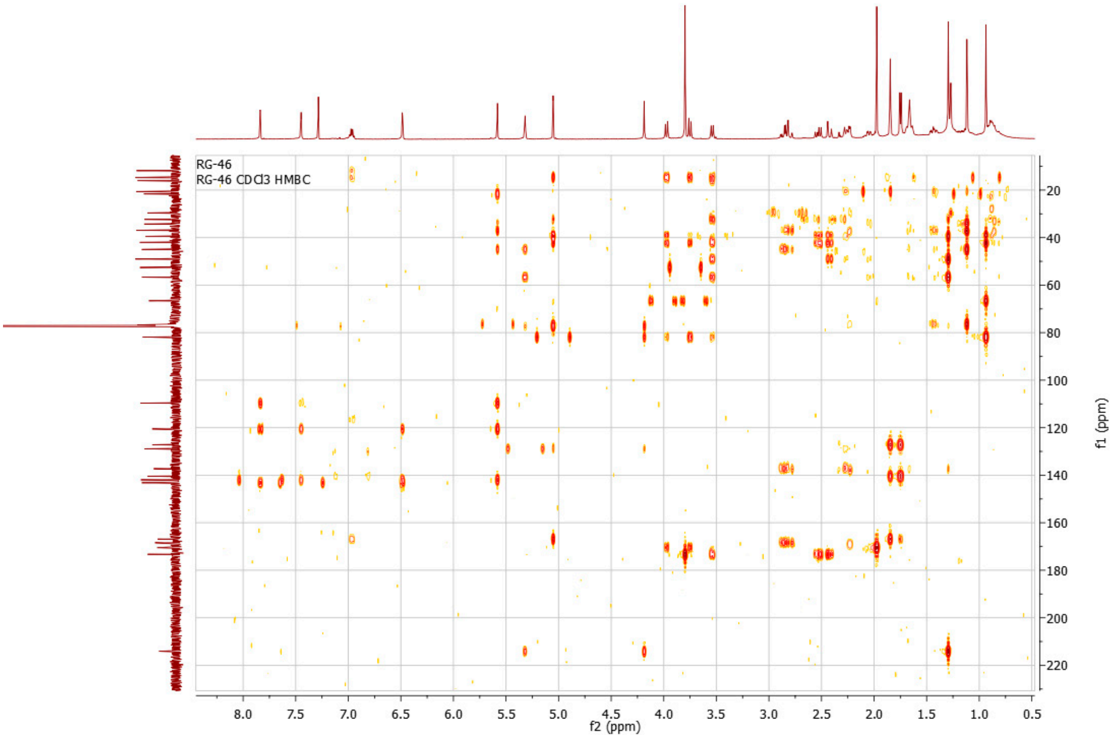

Figure S19. HMBC spectrum of compound **3** in $CDCl_3$.

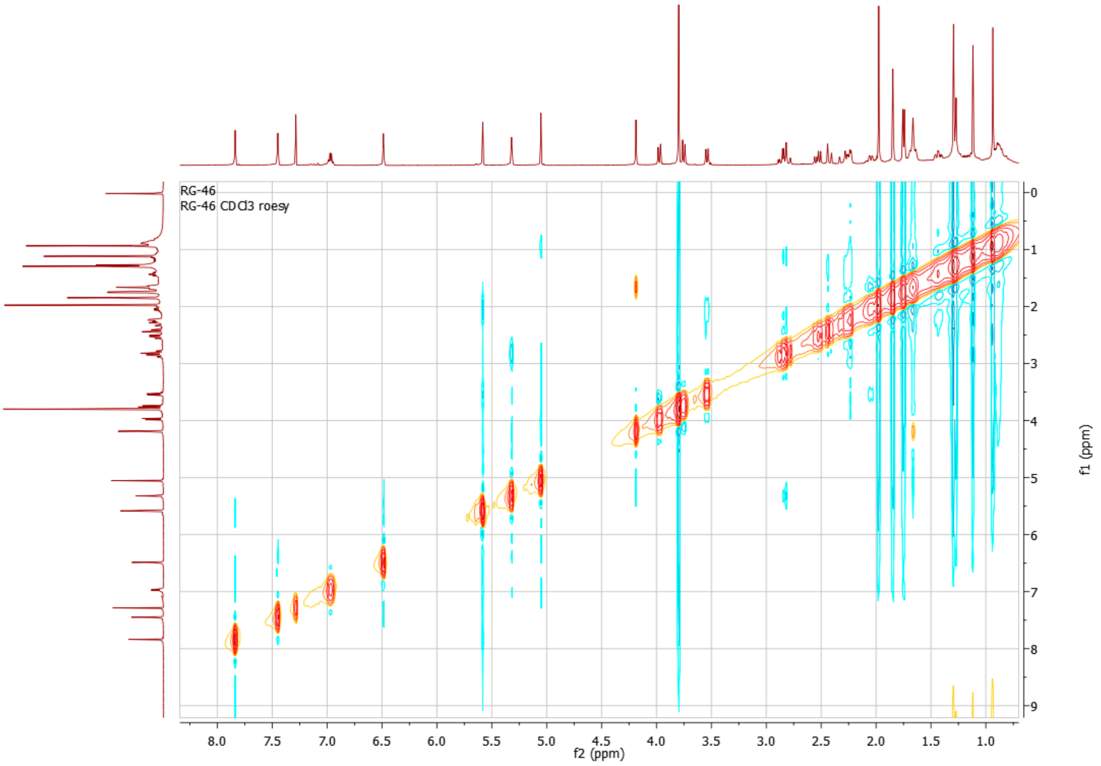

Figure S20. ROESY spectrum of compound **3** in $CDCl_3$.

Qualitative Analysis Report

**Data Filename**	140401ESIA13.d	**Sample Name**	RG-46
**Sample Type**	Sample	**Position**	
**Instrument Name**	Agilent G6230 TOF MS	**User Name**	KIB
**Acq Method**	ESI.m	**Acquired Time**	4/1/2014 10:45:24 AM
**IRM Calibration Status**	Success	**DA Method**	ESIN.m
**Comment**			

Sample Group **Info.**
Acquisition SW 6200 series TOF/6500 series
Version Q-TOF B.05.01 (B5125.1)

User Spectra

Fragmentor Voltage	Collision Energy	Ionization Mode
200	0	ESI

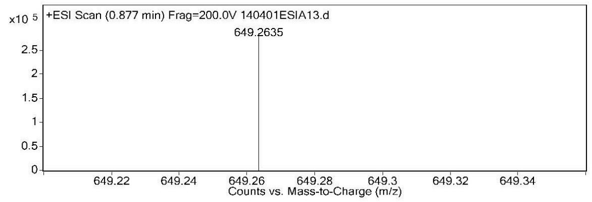

Peak List

m/z	z	Abund
1275.5356	1	372917.97

Formula Calculator Element Limits

Element	Min	Max
C	0	200
H	0	400
O	10	15
Na	1	1

Formula Calculator Results

Formula	CalculatedMass	Mz	Diff.(mDa)	Diff. (ppm)	DBE
C34 H42 Na O11	649.2625	649.2635	-1.0	1.5	13.5

--- End Of Report ---

Figure S21. HRESI-MS spectrum of compound **3**.

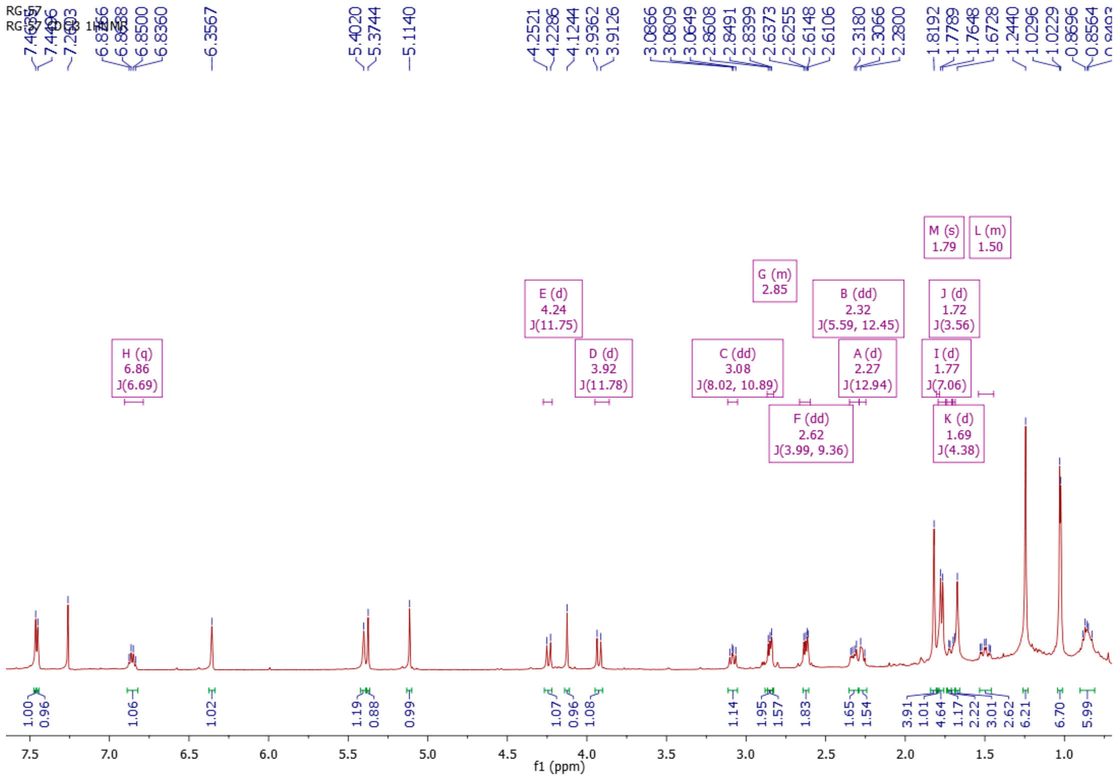

Figure S22. ^{1}H-NMR (500 MHz, $CDCl_3$) spectrum of compound **4**.

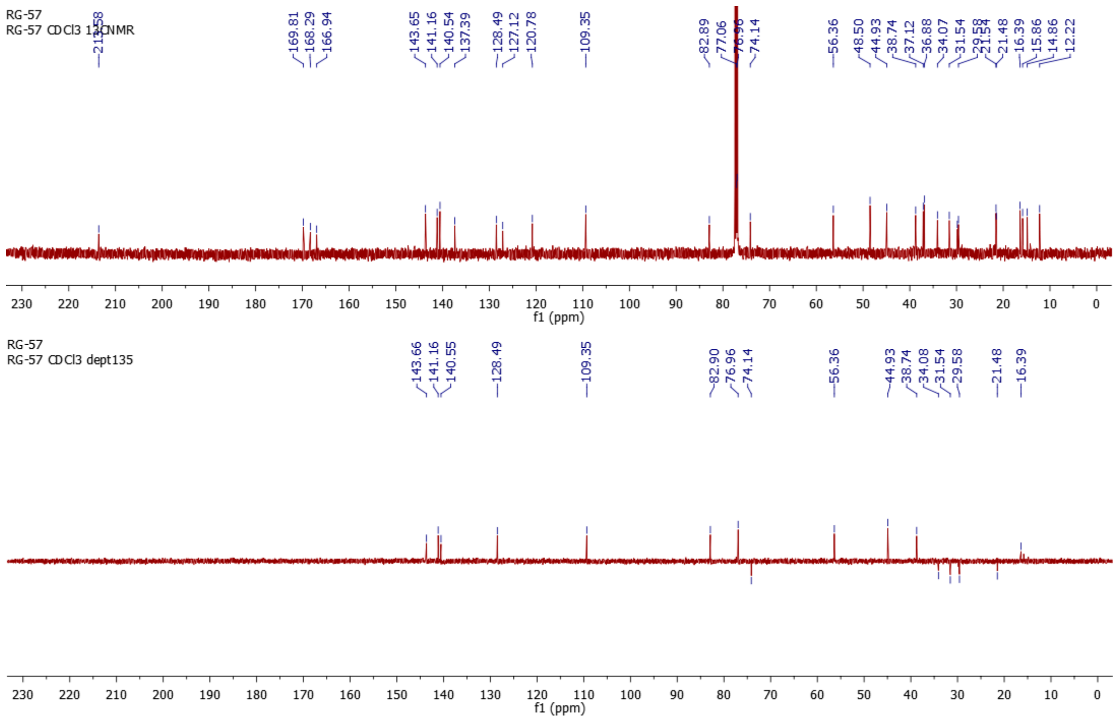

Figure S23. ^{13}C-NMR (125 MHz, $CDCl_3$) spectrum of compound **4**.

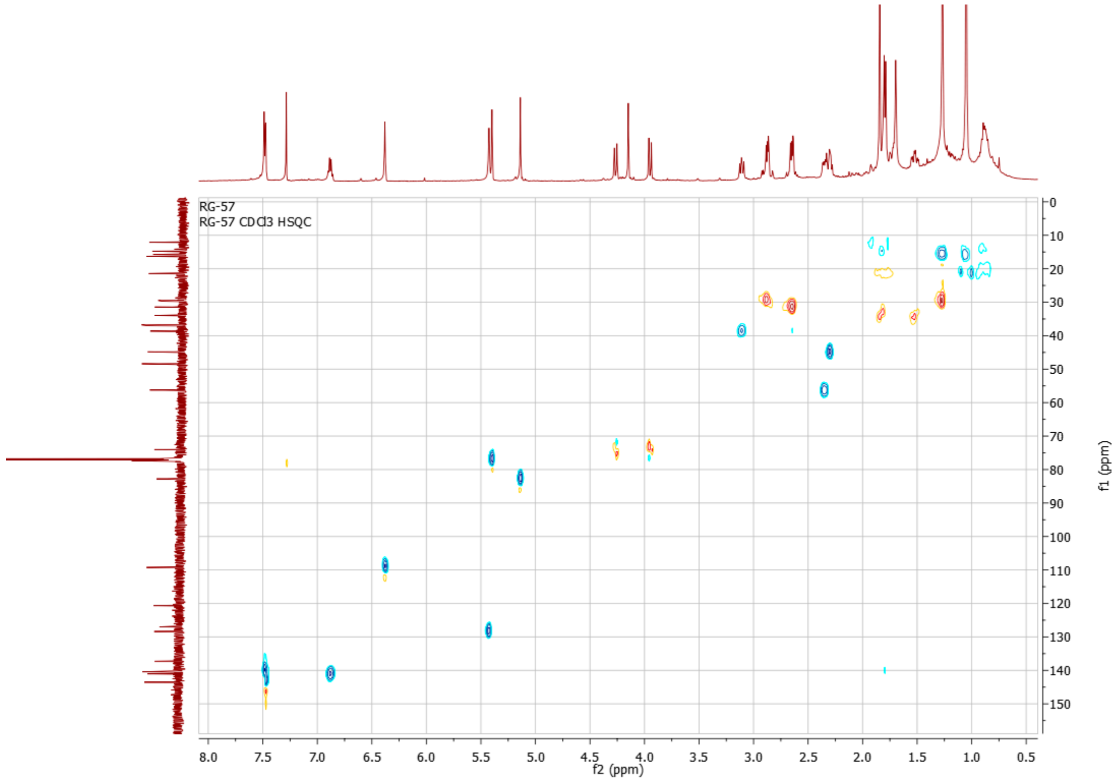

Figure S24. HSQC spectrum of compound **4** in $CDCl_3$.

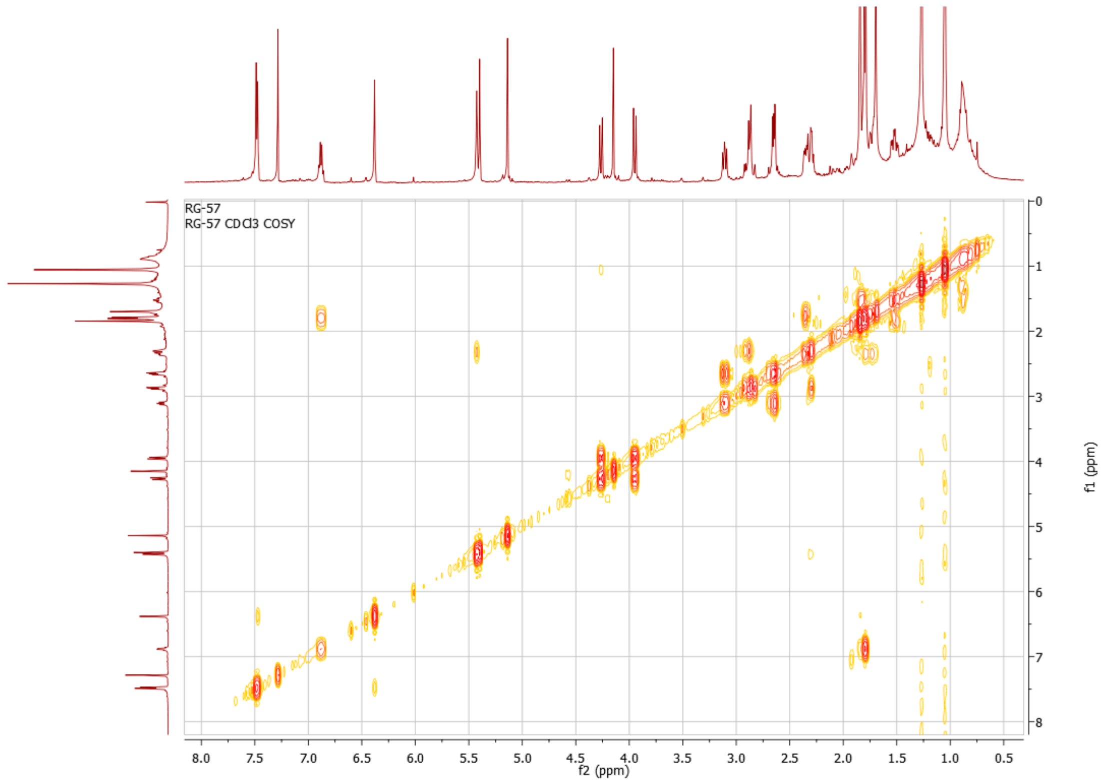

Figure S25. ^{1}H-^{1}H COSY spectrum of compound **4** in $CDCl_3$.

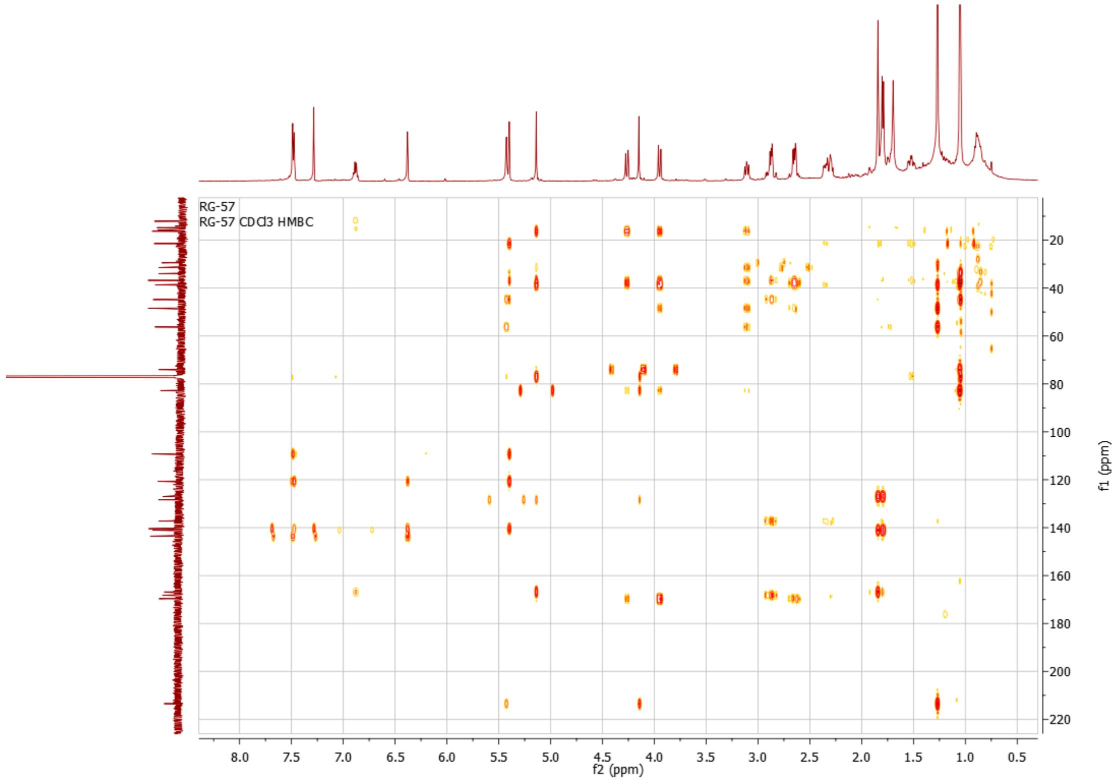

Figure S26. HMBC spectrum of compound **4** in $CDCl_3$.

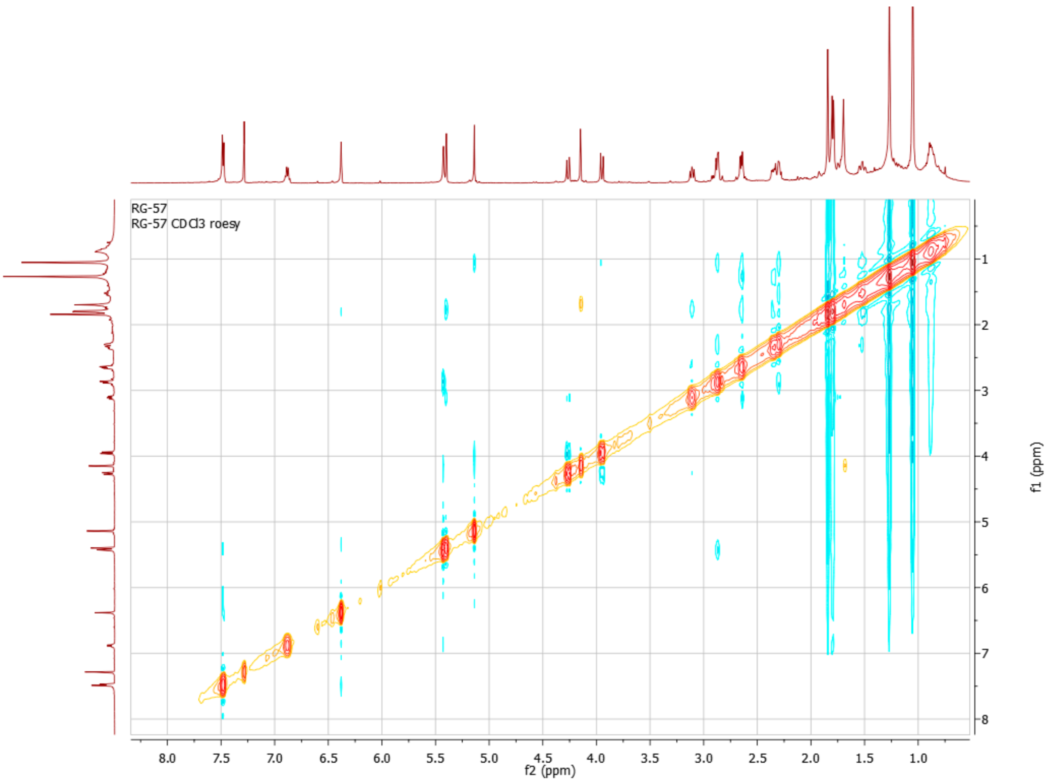

Figure S27. ROESY spectrum of compound **4** in $CDCl_3$.

Qualitative Analysis Report

**Data Filename**	140402ESIA6.d	**Sample Name**	RG-57
**Sample Type**	Sample	**Position**	
**Instrument Name**	Agilent G6230 TOF MS	**User Name**	KIB
**Acq Method**	ESI.m	**Acquired Time**	4/2/2014 11:39:54 AM
**IRM Calibration Status**	Success	**DA Method**	ESIN.m
**Comment**			

**Sample Group**		**Info.**
**Acquisition SW**	6200 series TOF/6500 series	
**Version**	Q-TOF B.05.01 (B5125.1)	

User Spectra

Fragmentor Voltage	Collision Energy	Ionization Mode
200	0	ESI

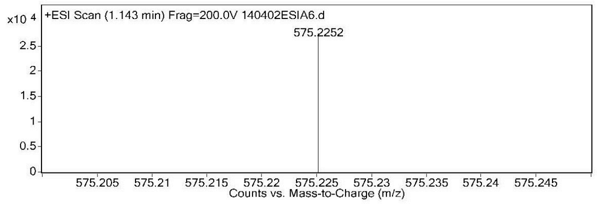

Peak List

m/z	z	Abund
607.252	1	299401

Formula Calculator Element Limits

Element	Min	Max
C	0	200
H	0	400
O	7	11
Na	1	1

Formula Calculator Results

Formula	CalculatedMass	Mz	Diff.(mDa)	Diff. (ppm)	DBE
C31 H36 Na O9	575.2257	575.2252	0.5	0.9	13.5

--- End Of Report ---

Figure S28. HRESI-MS spectrum of compound **4**.
